# Plantar fascia thickness in type 1 diabetes mellitus patients: Clinical associations and metabolic correlates

**DOI:** 10.1111/jdi.70310

**Published:** 2026-04-27

**Authors:** Giulia Casadei, Michele Baldari, Lorenzo Brognara, Enrico Saudelli, Simona Moscatiello, Sara Flamigni, Francesco Traina, Alexandro Paccapelo, Uberto Pagotto, Guido Di Dalmazi

**Affiliations:** ^1^ Division of Endocrinology and Diabetes Prevention and Care IRCCS Azienda Ospedaliero‐Universitaria di Bologna Bologna Italy; ^2^ Department of Medical and Surgical Sciences (DIMEC) Alma Mater Studiorum University of Bologna Bologna Italy; ^3^ Department of Biomedical and Neuromotor Sciences‐DIBINEM University of Bologna Bologna Italy; ^4^ Orthopaedic‐Traumatology and Prosthetic Surgery and Revisions of Hip and Knee Implants IRCCS Istituto Ortopedico Rizzoli Bologna Italy; ^5^ Epidemiology and Statistics IRCCS Azienda Ospedaliero‐Universitaria di Bologna Bologna Italy

**Keywords:** advanced glycation end products, diabetic neuropathies, type 1 diabetes mellitus

## Abstract

**Introduction:**

Diabetic peripheral neuropathy (DPN) is a severe complication of diabetes mellitus (DM). Measurement of plantar fascia thickness (PFT) by ultrasound has been proposed as an alternative index of tissue glycation and a marker for diabetes complications such as DPN.

**Research Design and Methods:**

A cross‐sectional study was conducted in 290 patients with type 1 DM (age 54 [20–87] years and diabetes duration 28 [1–79] years) and 41 healthy volunteers (age 49 [26–73] years). PFT was measured during outpatient clinical evaluation by means of ultrasound. Values were compared with sex, age, anthropometric parameters, advanced glycation end products (AGEs) measured by skin autofluorescence, and parameters derived from clinical evaluation of DPN.

**Results:**

Patients with type 1 DM had significantly thicker plantar fascia than controls (2.9 ± 0.6 mm vs. 2.4 ± 0.2 mm; *P* < 0.001); 199/290 (68.6%) patients with type 1 DM had altered PFT. In this population, male patients and those with worse glycemic control and longer duration of disease had a higher risk of having altered PFT. Higher PFT was significantly associated with reduced vibration sensitivity, higher levels of HbA1c and AGEs, presence of retinopathy and reduced renal function, and a worse diabetic foot risk classification. The use of AHCL integrated systems appeared to be associated with better plantar fascia outcomes.

**Conclusions:**

Our findings indicate that PFT showed a relationship with diabetes microvascular complications, glucometabolic compensation, and with the use of automated insulin delivery systems. PFT might be a useful tool in the assessment of patients with T1DM.

## Graphical abstract

Ultrasound‐measured plantar fascia thickness is associated with microvascular complications and glycemic control in individuals with type 1 diabetes, suggesting PFT as a potential non‐invasive tool for clinical assessment. In addition, improved thickness values were observed in patients using automated insulin delivery systems.

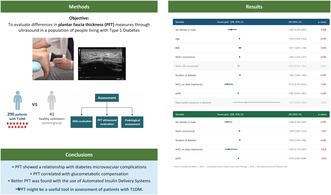

**Abbreviations**
AER: albumin escretion ratio
AGEs: advanced glycation end products
AGP: ambulatory glucose profile
AHCL: advanced hybrid closed loop
AU: arbitrary units
BMI: Body Mass Index
CV: coefficient of variation
DM: diabetes mellitus
DPN: diabetic peripheral neuropathy
GMI: glucose management indicator
HCL: hybrid closed loop systems
isCGM: intermittently scanned continuous glucose monitoring
LOPS: loss of protective sensation
PFT: plantar fascia thickness
ROS: reactive oxygen species
rtCGM: real time continuous glucose monitoring
SAF: SKIN autofluorescence
SMBG: self‐monitoring blood glucose
T1DM: type 1 diabetes mellitus
TAR: time above range
TBR: time below range
TIR: time in range
WBLT: weight‐bearing lunge test

## INTRODUCTION

Type 1 diabetes mellitus (T1D) is an autoimmune disorder characterized by increased blood glucose levels due to a deficiency in insulin secretion. Self‐monitoring blood glucose (SMBG) measurements or continuous glucose monitoring (CGM) and exogenous insulin therapy are the cornerstones of the treatment. If not properly treated, T1D could lead to severe micro‐ and macrovascular complications.

Chronic hyperglycemia leads to increased substrate flux through glycolysis and tricarboxylic acid cycle, generating through the electron transport chain increased potential difference across the inner mitochondrial membrane in order to produce ATP. However, the reduction of oxygen, the final electron acceptor in the chain, generates superoxide and reactive oxygen species (ROS). The increased production of ROS results in the formation of dicarboxyl products called advanced glycation end products (AGEs) through autoxidation of glucose. AGEs modify intracellular proteins, lipids, and nucleic acids through nonenzymatic reactions, eventually leading to neuronal inflammation and increased vascular permeability. The accumulation of AGEs has been recognized as an independent pathogenetic factor for the development of diabetic neuropathy (DN) by increasing inflammation of nerve fibers and interfering with axonal transport^1^, ^2^.

The AGE methylglyoxal has been found responsible also for hyperalgesia associated with DN^3^. The accumulation of AGEs in the extracellular matrix promotes their binding to collagen, whose fibers are the main component of the plantar fascia. This binding increases the density of the fascia with a consequent increase in the thickness of this tissue^4^.

A few data have been published until now on the plantar fascia thickness in patients with T1D. Moreover, no data in the literature have analyzed the potential impact of the different treatments of diabetes on plantar fascia thickness.

The first aim of the study was to evaluate the plantar fascia thickness in patients with T1D under different treatments. The second aim was to investigate the association between plantar fascia thickness and parameters of metabolic control of diabetes.

## RESEARCH DESIGN AND METHODS

### Subjects

We enrolled 290 consecutive adult (≥18 years) patients with T1D who were evaluated from February 2023 to February 2024 at the Endocrinology and Diabetes Prevention and Care Unit, IRCCS S. Orsola Polyclinic (Bologna). We excluded patients with uncertain diagnosis of autoimmune diabetes, inability to walk or perform the study protocol, and pregnant women.

We also recruited 41 healthy volunteers from the University Podiatry Clinic of the Rizzoli Orthopedic Institute (Bologna) as controls.

### Podiatric assessment and advanced glycation end products (AGEs) measurement

According to the current guidelines, all patients underwent a comprehensive diabetic foot assessment. Evaluation of foot sensitivity was performed by a single operator (G.C.), by using a Semmes‐Weinstein monofilament and a biothesiometer. The monofilament was used to test three different sites on both feet. Protective sensation was defined present at each site if the patient answers correctly on two out of three applications. The biothesiometer (model “Ultrabiotesiometro,” Me.Te.Da S.r.l., Ascoli‐Piceno, Italy) was used to test neural function by applying vibrations of progressively increasing intensity to the foot, until the detection of the vibration by the patient or the maximum of 50 V. Detection of vibration at values greater than 25 V in the dorsal area of the big toe was considered a predictor of the risk of foot ulceration, as it has been associated with distal symmetric polyneuropathy^5^. Patients showing alterations in at least one of those tests were defined as having diabetic peripheral neuropathy (DPN) due to loss of protective sensation (LOPS).

The ankle range of motion was measured using the weight‐bearing lunge test (WBLT). To perform this test, patients were asked to stand in front of the wall with their feet on a measuring tape placed on the floor, starting at a distance from the wall of 10 cm. The distance between the big toe and the wall was recorded and expressed in centimeter, at the point where the patients could touch the wall with their knee without lifting the heel from the floor^6^, ^7^.

Peripheral artery disease (PAD) was assessed using ankle brachial index (ABI). Values of ABI <0.9 were considered pathological. Finally, patients were stratified using the IWGDF Risk Stratification System. Participants without LOPS and PAD were classified as IWGDF risk 0 (no risk of ulceration), whereas participants with either LOPS or PAD, in the absence of additional risk factors, were assigned to IWGDF risk 1 category (low risk of ulceration). Patients with LOPS or PAD and digital deformities were considered as IWGDF risk 2 (moderate risk of ulceration), while those with either LOPS or PAD and a history of foot ulcer or lower extremity amputation were classified as IWGDF risk 3 (high risk of ulceration).

Skin autofluorescence was used to assess AGEs levels by asking the patient to place the volar part of the forearm on the AGE Reader device AGE Reader MU, Diagnoptics, Groningen, the Netherlands. Approximately 4 cm^2^ of skin of this area was illuminated with an excitation light source from the AGE Reader with a peak wavelength of 370 nm. The device estimated skin AGEs based on the emission and reflection spectrum, which was converted through software into numerical values reported in arbitrary units (AU). Thus, an elevated skin autofluorescence score corresponded to high tissue AGEs levels.

Plantar fascia thickness (PFT) was evaluated by ultrasound (MyLab™ Sigma, ESAOTE S.p.A, Genoa, Italy) using a 12‐MHz linear array transducer, by placing the probe along the longitudinal axis of the plantar fascia at 1 cm distal from its origin from calcaneus, while the patient was lying prone.

### Anthropometric, biochemical and clinical data

Anthropometric and biochemical data were collected for each patient at the time of the podiatric assessment. Mean and standard deviation of all values of glycated hemoglobin (HbA1c) of the previous 3 years were calculated in patients with at least three measurements. Prevalence of main diabetes‐related complications and the onset date were collected, together with associated autoimmune disorders. Diabetic retinopathy was defined as a neurovascular complication of diabetes characterized by microaneurismal in the earliest stage, followed by retinal hemorrhages and ischemia which through the release of proliferation factors lead to formation of neo‐vessels: according to the 2026 American Diabetes Association Standards of Care, the presence of neo‐vessels divides the classification in non‐proliferative and proliferative retinopathy, where intravitreal hemorrhage and retinal detachment may be potential causes of sudden blindness. Type of glucose monitoring was recorded and classified into self‐monitoring blood glucose (SMBG), intermittently scanned and real‐time continuous glucose monitoring (isCGM and rtCGM, respectively). Glucose metrics like time in range (TIR), which is the percentage of time in the 70–180 mg/dL glycemia during the day, time above range (TAR, the percentage above 180 mg/dL), time below range (TBR, the percentage under 70 mg/dL), glucose management index (GMI, also known as the approximate A1C level based on sensor glycemias) and coefficient of variation (CV, an indicator of glucose variability) were recorded according to the current guidelines^8^. Each of the metrics was analyzed for the 14 and 90 days before the evaluation. We also recorded whether patients were under multiple daily insulin injections (MDI), stand‐alone insulin pump, hybrid closed loop systems (HCL), or advanced HCL (AHCL). Duration of use of the specific treatment was calculated from the time of first placement to the time of plantar fascia evaluation.

### Statistical analysis

Patient demographic and clinical characteristics were reported as frequencies and percentages for categorical variables and as mean ± standard deviation and range for continuous variables. The 97.5^th^ percentile of the healthy control group was chosen as the upper limit for normal plantar fascia thickness after the bootstrapping procedure. The Pearson's chi‐squared test, the Fisher's exact test, and the *t*‐test were used to compare groups. The univariable and multivariable logistic regression were used to investigate the association between patient characteristics and altered plantar fascia thickness (≥2.7 mm). The backward stepwise method was used as the selection method in the multivariable model. The odds ratio (OR) was computed together with their 95% confidence intervals (95% CI). The Pearson's correlation coefficient (*r*) was computed.

The *P*‐value was considered significant when <0.05 for two‐tailed tests. Statistical analysis was performed using IBM SPSS Statistics for Windows software, Version 29.0 (Armonk, NY: IBM Corp).

## RESULTS

Descriptive data of patients with T1D and controls are shown in Table 1. Female sex was evenly distributed (44.8% in patients with T1D vs. 53.7% in controls; *P* = 0.318), whereas patients were older than controls (54.2 ± 16.7 vs. 44.7 ± 12.6 years; *P* < 0.001). Patients showed a mean HbA1c of 57 ± 11.6 mmol/mol in a mean duration of disease of 27.6 ± 14.5 years. The majority of the patients monitored their blood glucose levels by isCGM (*n* = 156, 53.8%), 69 patients (23.8%) used SMBG with multi‐injective therapy whereas rtCGM was used by 65 patients (22.4%) either with MDI (*n* = 29, 10%) or AHCL/HCL (*n* = 36, 12.4%). As widely reported in literature, all CGM‐derived metrics were better in the AHCL group rather than in the non‐AHCL one: TIR 72.4% ± 14.6 vs. 56.3% ± 17.5, TAR 26.1% ± 14.8 vs. 39.3% ± 19.4, TBR 1.8% ± 2.5 vs. 5.0% ± 6.8, CV 30.7% ± 6.8 vs. 37.5% ± 7.0.

**Table 1:** Anthropometric, clinical and laboratory characteristics of the whole study group divided by T1DM patients and healthy volunteers

	Type 1 diabetes	Controls	Total	*P*‐value
Demographic and anthropometric characteristics of the two populations	*n* = 290	*n* = 41	*n* = 331	
Sex: female, *n* (%)	130 (44.8%)	22 (53.7%)	152 (45.9%)	0.318
Age, years	54.2 ± 16.7	44.7 ± 12.6	53.0 ± 16.5	<0.001
Diabetes duration, years	27.6 (1–79) ± 14.5			
BMI, kg/m^2^	25.1 (17.6–40.7) ± 4.0	23.5 ± 3.1		
Waist circumference, cm	91.5 (64–128) ±12.5		^†^	
Smoker status				
Nonsmokers	165 (56.9%)	21 (51.2%)	186 (56.2%)	
Previously smoking	69 (23.8%)	9 (22.0%)	78 (23.6%)	
Actively smoking	56 (19.3%)	11 (26.8%)	67 (20.2%)	
Alcoholic habit				
Abstemious	68 (23.4%)	0 (0.0%)	68 (20.5%)	
2–3 times a day	211 (72.8%)	38 (92.7%)	249 (75.2%)	
>5 times a day	11 (3.8%)	3 (7.3%)	14 (4.2%)	
Physical activity				
No, *n* (%)	132 (45.5%)	15 (36.6%)	147 (44.4%)	
Moderate, *n* (%)	116 (40.0%)	22 (53.7%)	138 (41.7%)	
Intense, *n* (%)	42 (14.5%)	4 (9.8%)	46 (13.9%)	
Laboratory characteristics				
HbA1c, mmol/mol	57.1 (25–107) ± 11.6			
Mean HbA1c, mmol/mol	58.8 (33.4–112) ±10.3			
HbA1c SD, mmol/mol	5.17 (1.0–44.3) ± 3.75			
Creatinine, mg/dl	0.9722 (0.53–11.10) ± 0.8			
Renal filtration, mL/min	87.6 ± 22.2			
Microalbuminuria, μg/min (median and interquartile range)	5.0 (4.0–9.5)			
LDL, mg/dl	94.8 (21–185) ± 26.9			
GOT, UI/L	24.0 (11–106) ±10.1			
GPT, UI/L	20.7 (5–134) ±12.6			
TSH, μU/mL	3.0 (0.2–74) ±6.0			
Device				
MDI/micro SMBG, *n* (%)	69 (23.8%)			
MDI/micro isCGM, *n* (%)	156 (53.8%)			
MDI/micro rtCGM, *n* (%)	29 (10.0%)			
AHCL/HCL/PLGS, *n* (%)	36 (12.4%)			
Duration from sensor positioning, years	3.6 (0–17.4) ±2.4			
TIR, %	59 (0–94) ±18			
TAR, %	37 (1–100) ±19			
TBR, %	4.4 (0–68) ±6.4			
CV, %	36.2 (7–57) ± 7.5			
Duration from micro positioning, years	5.9 (0.1–34.6) ±7.3			
TDD/kg ratio, UI/kg	0.55 (0.12–1.42) ±0.19			
Associated diseases and complications^‡^				
Celiac disease, *n* (%)	24 (8.6%)			
Thyroid disease, *n* (%)	109 (37.6%)			
Autoimmune thyroiditis, *n* (%)	85 (45.9%)			
Hypothyroidism, *n* (%)	68 (39.5%)			
Hyperthyroidism, *n* (%)	6 (3.5%)			
Hypertension, *n* (%)	102 (35.4%)			
Cardiovascular disease, *n* (%)	33 (11.7%)			
Diabetic nephropathy, *n* (%)	40 (13.9%)			
Nephropathy years, years	5.5 (0–18.4) ±5.4			
Diabetic retinopathy, *n* (%)	100 (35.2%)			
Retinopathy years, years	8.1 (0–26.2) ±5.5			

Data are expressed as mean with minimum and maximum range in parentheses and standard deviation, or as frequencies; exception was made for the microalbuminuria variable which was not normally distributed.

^†^ Waist circumference was not measured in the control group.

^‡^ In T1DM population, data about celiac disease were found in 279 patients, data about thyroid disease were found in 290 patients, data about autoimmune thyroiditis were found in 185 patients, data about hypothyroidism were found in 172 patients, data about hyperthyroidism were found in 172 patients, data about hypertension were found in 288 patients, data about cardiovascular disease were found in 281 patients, data about diabetic nephropathy were found in 287 patients, data about diabetic retinopathy were found in 287 patients.

BMI, body mass index; CSII, continuous subcutaneous insulin infusion; CV, coefficient of variation; GOT/GPT, glutamyl oxaloacetic/glutamyl pyruvic aminotransferase; HbA1c, glycated hemoglobin; isCGM, intermittently scanned continuous glucose monitoring (device); MDI, multiple daily injections; rtCGM, real‐time continuous glucose monitoring (device); SD, standard deviation; SMBG, self‐monitoring of plasma glucose; TAR, time above range; TBR, time below range; TDD, total daily dose; TIR, time in range; TSH, thyroid‐stimulating hormone.

**Figure 1:**
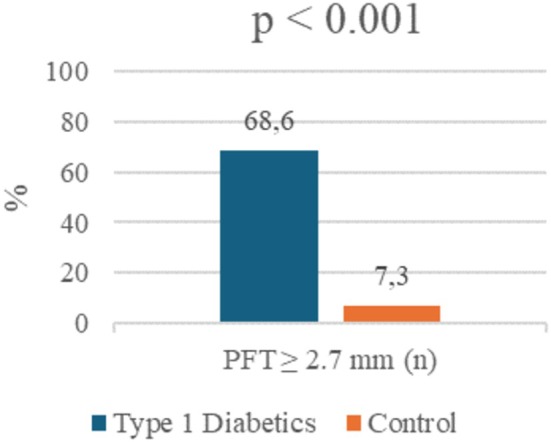
Proportion of patients with altered plantar fascia thickness in the T1DM and control groups. PFT, Plantar fascia thickness.

Data on podiatric assessment and AGEs are shown in Table 2. The IWGDF Risk Stratification System identified 23 patients (7.9%) at risk 3, 10 (3.5%) at risk 2, 102 (35.2%) at risk 1, and 155 (53.4%) at risk 0: a correlation between plantar fascia thickness and this risk was found (*r* = 0.287, *P* < 0.001). Vibratory sensitivity, to mean a biothesiometer outcome >25 V, was altered in 76 patients (26.2%), with LOPS in 9 (3.1%) subjects. ABI identified 24 patients (8.3%) at risk of PAD, whereas altered ankle joint mobility (Lunge test <10 cm) was recorded in 181 (62.4%) subjects. All previous tests showed a statistically significant alteration in the plantar fascia thickness (Table S1). Among the population, only two patients had an absent pedideal pulse, so it was not possible to study a correlation, and so was for the monofilament test where only nine patients had a negative touch test and apparently no correlation was found. As another diabetes‐related complication, the presence of retinopathy, found in 101 patients (35.2%), was correlated with a thicker plantar fascia (3.1 ± 0.6 vs. 2.8 ± 0.5, *P* < 0.001), as shown in Table S1; another correlation was found between PFT and the presence of cardiovascular disease such as stroke or myocardial infarction (3.1 ± 0.5 vs. 2.9 ± 0.6, *P* = 0.018) (Table S2). As for the comorbidities, higher PFT was associated with the presence of hypertension (3.0 ± 0.6 vs. 2.8 ± 0.6, *P* = 0.022) but not with obesity (*P* = 0.453) or LDL values >100 mg/dL (*P* = 0.420). Despite some comorbidities were associated with PFT in this population, we decided to not include them in the final model, due to the risk of reducing the statistical power of the model by increasing too much the number of co‐variates in this relatively small cohort of patients. The mean plantar fascia thickness was 2.9 ± 0.6 mm at 1 cm proximal to the insertion point, which was significantly higher than controls (2.4 ± 0.2 mm; *P* < 0.001). The significant differences were confirmed also in analysis conducted separately by sex, resulting in a thickening of plantar fascia both in the male (3.0 mm ± 0.6) and in the female subgroups of patients (2.8 mm ± 0.5) (*P* < 0.001 in both populations). Moreover, no difference in plantar fascia thickness was detected between males and females in control population (males 2.4 ± 0.18 mm vs. females 2.4 ± 0.17 mm, *P* = 0.165). No correlation was found between plantar fascia thickness and age in controls (*r* = 0.224, *P* = 0.159). Therefore, we set the upper limit of normal plantar fascia thickness at 2.7 mm (97.5^th^ percentile of the values of control group after bootstrap procedure) for the whole population. The proportion of patients with T1D with altered plantar fascia thickness (≥2.7 mm) was 68.6% (*n* = 199) (Figure 1).

**Table 2:** Data on podological assessment

	Type 1 diabetes	Controls	Total	*P*‐value
PFT, mm	2.9 ± 0.6	2.4 ± 0.2	2.8 ± 0.6	<0.001
PFT ≥2.7, *n* (%)	199 (68.6%)	3 (7.3%)	202 (61.0%)	<0.001
AGEs, AU	2.4 ± 0.5	1.9 ± 0.3	2.4 ± 0.6	<0.001
IWGDF risk classification				
0, *n* (%)	155 (53.4%)			
1, *n* (%)	102 (35.2%)			
2, *n* (%)	10 (3.4%)			
3, *n* (%)	23 (7.9%)			
Lunge test				
>10 cm, *n* (%)	109 (37.6%)			
5–10 cm, *n* (%)	114 (39.3%)			
0–5 cm, *n* (%)	67 (23.1%)			
Ankle‐Brachial Index				
>0.9 mmHg/mmHg, *n* (%)	149 (51.4%)			
0.7–0.9 mmHg/mmHg, *n* (%)	19 (6.6%)			
0.5–0.7 mmHg/mmHg, *n* (%)	5 (1.7%)			
>1.3 mmHg/mmHg, *n* (%)	117 (40.3%)			
Biothesiometer				
0–20 V, *n* (%)	144 (49.7%)			
20–25 V, *n* (%)	70 (24.1%)			
>25 V, *n* (%)	76 (26.2%)			
Pedideal pulse				
No, *n* (%)	2 (0.7%)			
Yes, *n* (%)	288 (99.3%)			
Deformities				
No, *n* (%)	211 (72.8%)			
Yes, *n* (%)	79 (27.2%)			
Altered plantar pressure				
No, *n* (%)	188 (64.8%)			
Yes, *n* (%)	102 (35.2%)			
Monofilament test				
Absent response, *n* (%)	9 (3.1%)			
Present, *n* (%)	281 (96.9%)			

Data are expressed as mean with standard deviation, or as frequencies with percentage in parenthesis.

Abbreviations: AGE, advanced glycation end products; AU, arbitrary units; IWGDF, International Working Group on the Diabetic Foot; V, volts.

Advanced glycation end products (AGEs) were significantly higher in T1D patients than controls (2.4 ± 0.5 AU in T1D *vs* 1.9 ± 0.3 AU in controls; *P* < 0.001). Among T1DM‐affected patients, higher AGE levels were correlated with higher TAR values (*r* = 0.146, *P* < 0.05), while there was no other correlation with the other CGM parameters. A significant correlation between AGE levels and plantar fascia thickness was also found (*r* = 0.418, *P* < 0.001). This correlation was stronger in the patient group rather than in the control one, being *r* = 0.355 (*P* < 0.001) and *r* = 0.281 (*P* = 0.076), respectively.

According to univariable analysis, a significant association was found between higher plantar fascia thickness and male sex (*P* = 0.008), age (*P* < 0.001), BMI (*P* = 0.045), HbA1c (*P* < 0.001), duration of diabetes (*P* < 0.001), lower EGFR (*P* < 0.001), and use of diabetes treatments other than AHCL (*P* = 0.001) (Table 3). Multivariable analysis confirmed the significant association between plantar fascia thickness and male sex, HbA1c, duration of diabetes, EGFR, and use of AHCL as independent factors (Table 3). Finally, no correlation was found between CGM parameters or the use of CGMs itself and plantar fascia thickness.

**Table 3:** Risk factors for high plantar fascia thickness

	OR (95% CI)	*P*‐value
Univariate analysis		
Sex (female vs. male)	**0.547 (0.350–0.855)**	**0.008**
Age	**1.039 (1.024–1.054)**	**<0.001**
BMI	**1.071 (1.001–1.146)**	**0.045**
HbA1c (mmol/mol)	**1.045 (1.019–1.071)**	**<0.001**
HbA1c standard deviation (mmol/mol)^†^	0.975 (0.914–1.041)	0.457
Duration of diabetes	**1.062 (1.040–1.085)**	**<0.001**
AHCL (vs. other treatments)	**0.310 (0.152–0.633)**	**0.001**
EGFR	**0.967 (0.953–0.981)**	**<0.001**
Nephropathy (presence vs. absence)	1.437 (0.670–3.084)	0.352
Multivariate analysis		
Sex (female vs. male)	**0.306 (0.160–0.583)**	**<0.001**
HbA1c (mmol/mol)	**1.040 (1.009–1.072)**	**0.011**
Duration of diabetes	**1.056 (1.030–1.082)**	**<0.001**
AHCL (vs. other treatments)	**0.393 (0.170–0.907)**	**0.029**
EGFR	**0.978 (0.960–0.996)**	**0.019**

Results of univariate and multivariate analyses. After clustering all the risk factors found in the univariate analysis, selected variables in the multivariate analysis were processed, and sex, HbA1c, duration of diabetes, AHCL, and EGFR were found as truly predictive risk factors. Significant differences are highlighted in bold.

^†^ Standard deviation regarding mean of 3 years of HbA1c results prior to visit.

AHCL, Advanced Hybrid Closed Loop; BMI, body mass index; CI, confidence interval; EGFR, Estimated Glomerular Filtration Rate; HbA1c, glycated hemoglobin; OR, odds ratio.

## DISCUSSION

This study highlights the relationship between plantar fascia thickness, AGEs, and clinical parameters, such as metabolic control of diabetes, duration of diabetes, EGFR, and podiatric parameters of diabetic foot risk. Moreover, the use of AHCL emerged as an independent protective factor for plantar fascia thickening in patients with T1D.

Mean plantar fascia thickness was 2.9 ± 0.6 mm in T1DM patients. This outcome is comparable to that obtained in the literature in other studies conducted in Caucasian populations composed of Italians, the same population that was analyzed in ours^9^. When compared to control subjects, the plantar fascia thickness was altered in the majority of the patients (68.6%)^4^. To our knowledge, the present study is the first to investigate the relationship between plantar fascia with distal symmetric polyneuropathy, limited joint mobility, and the IWGDF Risk Stratification System. Glycation of collagen in tissue seems to lead to greater tendon stiffness. Several studies previously agreed that limited dorsiflexion ankle mobility leads to an increased forefoot pressure, walking impairments, and postural instability^10^. Therefore, these data support the evaluation of plantar fascia thickness as a biomarker of complications during the screening and assessment of patients with T1D^11^, ^12^.

Our results also confirm recent findings on the association between plantar fascia thickness and higher levels of HbA1c, remarking the link between poor metabolic control of diabetes and non‐enzymatic glycation of connective tissue^13^. Indeed, AGE levels were also correlated to plantar fascia thickness, with a stronger association in patients with T1D rather than controls. These data support the idea that measurement of AGEs and plantar fascia thickness may provide similar information on the status of non‐enzymatic glycation, therefore providing complementary data to the evaluation of HbA1c and glucose metrics.

The use of ambulatory glucose profile (AGP) reports, showing a pattern of individual glycemic trends related to the last 14 days, could be useful as a short‐term disease assessment tool, with predictive value on microvascular complications, particularly regarding TIR and CV^14^.

HbA_1c_, reflecting average glycemic exposure over approximately three months, is the established gold standard for assessing glycemic control. Reductions in HbA_1c_ are associated with a significant decrease in the risk of both macrovascular and microvascular complications in individuals with T1D^15^.

In an RCT conducted on 1,440 participants over 10 years, the difference in mean TIR between those developing and not developing a microvascular outcome was 10–12%, which corresponds to a difference of about 2.5 h per day spent in target and an HbA1c difference of 1.0–1.4%^16^. However, whereas glucose metrics may offer a partial and short‐term insight of the patient's blood glucose trends, HbA1c may be unreliable in pathological comorbidities such as anemia, hemoglobinopathies, or iron deficiency.

Evaluation of PFT^12^ and AGEs^17^ may offer a long‐term picture of the disease, likely being the result of damage accumulation during the course of the disease, as supported by the association between duration of diabetes and PFT in our multivariate analysis. Moreover, unlike HbA1c, these parameters may be unrelated to other concomitant clinical conditions and considered as a more reliable data in the prediction of diabetes‐related complications.

The multivariate analysis also highlighted the relationship between plantar fascia thickness and male sex, EGFR, and use of AHCL.

In the control group, no differences were found between PFT in the male and female population, contrastingly from what is evidenced by the literature^18^. These conflicting data may be explained by the fact that our ultrasonographic evaluation was conducted on the plantar fascia at 1 cm distal to the Achilles tendon insertion on the calcaneus, whereas the correlation in the literature was found at 1 cm proximal to this anatomical point.

However, we also found male sex as an independent risk factor of PFT thickening in the T1DM population. We can justify this finding by assuming that there are gender‐related processes that can enhance the glycation of collagen fibers. Indeed, studies in the literature conducted on rats seemed to show greater production of AGEs in the male sex, supposing a better biochemical protection in the female gender^19^. Plantar fascia thickness and high AGE levels were inversely correlated with reduced EGFR, whereas the two of them seemed not to be linked with the presence of nephropathy, in contrast to what has been reported in the literature. As stated by the National Kidney Foundation, nephropathy is defined not only as reduced glomerular filtration rate < 60 mL/min but also as the persisting evidence of kidney damage for at least 3 months, namely this as the presence of albuminuria or traces of blood in urine, instrumental findings such as pathological changes detectable by renal ultrasound or histological in renal biopsy. The fact that a relationship with renal filtrate, but not with nephropathy more broadly intended, was observed might suggest altered plantar fascia thickness (PFT ≥2.7 mm) in ultrasound evaluation as a more reliable marker of declining renal function, where albuminuria has not yet developed or is not found to be present. Such speculation may support ultrasonographic assessment of plantar fascia as a non‐invasive, reliable and cost‐effective imaging method for the evaluation of microvascular complications. These results are in line with previous studies that have demonstrated the correlation between the PFT and different comorbidities such as early renal dysfunction in T1DM^11^, but more studies are needed to confirm this hypothesis.

Additionally, this is the first study investigating the correlation between PFT and treatment of T1D, highlighting that the use of AHCL is associated with lower plantar fascia thickness when compared with all other therapy management modalities (HCL, PLGS, multiple daily insulin injections). Importantly, the use of AHCL was an independent protective factor independent of other associated factors mentioned previously. As widely established in the literature, AHCL systems are associated with better glycemic control and reduced glucose variability^20^. Although, to our knowledge, no studies supported the basis of a link between glycemic variability or hypoglycemia and plantar fascia thickening or AGE formation, a systematic review showed a relationship with ROS production, which is the basis for AGE formation^21^. We may speculate that AHCL could reduce AGE formation also through reduction of hypoglycemia and glycemic variability. This trait sheds light on a possible future field of research, such as investigating correlations between AHCL, AGP parameters, and PFT on a larger cohort.

A limitation recognized in this study is its cross‐sectional nature; therefore, no data on PFT have been collected during the follow‐up. Nevertheless, considering that the modifications in the plantar fascia due to non‐enzymatic glycation are expected to be slow, a prospective design could have been difficult to perform. The association with established predictive markers of complications of diabetes supports the strength of these results also in a cross‐sectional setting. The strengths of the study are the presence of a control group, allowing the generation of normative data, and the reliability of the data, since all the podiatric procedures, including the evaluation of the plantar fascia thickness in patients and controls, were performed by the same operator.

## CONCLUSIONS

The measurement of PFT is a useful tool in the assessment of patients with T1D, showing an independent association with several diabetes‐related complications. Moreover, the potential independent protective role of AHCL on thickening of plantar fascia deserves particular interest and should be further investigated in future studies.

## DISCLOSURE

The authors declare no conflict of interest.

Approval of the research protocol: The research project has been approved by Ethic Committee Area Vasta Emilia‐Centro and it conforms to the provisions of the Declaration of Helsinki in 1995 (as revised in Fortaleza, Brazil, October 2013).

Informed consent: All subjects gave informed consent and patient anonymity is guaranteed to be preserved.

Approval date of Registry and approval registration number of the study: The study protocol was approved by the ethical committee (1008/2021/Oss/AOUBo).

Animal Studies: N/A.

## Supporting information


**Table S1.** Correlation between diabetic complications and PFT.
**Table S2**. Correlation between comorbidities and PFT.

## Data Availability

The data that support the findings of this study are available on request from the corresponding author. The data are not publicly available due to privacy or ethical restrictions.
