# Supplementary material for: Plantar fascia thickness in type 1 diabetes mellitus patients: Clinical associations and metabolic correlates
Source: J Diabetes Investig. 2026 Apr 27;17(7):1210–7. doi: 10.1111/jdi.70310 (PMC13327322; doi:10.1111/jdi.70310)
Supplement: Supplementary file 1 — Table S1. Correlation between diabetic complications and PFT. Table S2. Correlation between comorbidities and PFT. [file JDI-17-1210-s001.docx]

**Supplementary Table 1: correlation between diabetic complications and PFT**

| **Presence of retinopathy** | **N (%)** | **PFT (mm)** | **P value** |
| --- | --- | --- | --- |
| Retinopathy | 101 (35.2%) | 3.1 ± 0.6 | **<0.001** |
| No retinopathy | 186 (64.8%) | 2.8 ± 0.5 |  |
| Total | 287 (100%) | 2.9 ± 0.6 |  |
| **Biothesiometer** |  |  |  |
| 0-20 | 144 (49.7%) | 2.7 ± 0.5 | **<0.001** |
| 20-25 | 70 (24.1%) | 3.0 ± 0.5 |  |
| >25 | 76 (26.2%) | 3.2 ± 0.6 |  |
| Total | 290 (100.0%) | 2.9 ± 0.6 |  |
| **Lunge test** |  |  |  |
| 10 | 137 (41.4% | 2.6 ± 0.6 | **<0.001** |
| 5-10 | 127 (38.4%) | 3.0 ± 0.5 |  |
| 0-5 | 67 (20.2%) | 3.0 ± 0.6 |  |
| Total | 331 (100.0%) | 2.8 ± 0.6 |  |
| **Ankle-Brachial Index** |  |  |  |
| >0.9 | 190 (57.4%) | 2.7 ± 0.5 | **<0.001** |
| 0.7-0.9 | 19 (5.7%) | 3.1 ± 0.6 |  |
| 0.5-0.7 | 5 (1.5%) | 3.1 ± 0.2 |  |
| >1.3 | 117 (35.3%) | 2.9 ± 0.6 |  |
| Total | 331 (100.0%) | 2.8 ± 0.6 |  |
| **Presence of Deformities** |  |  |  |
| Yes | 79 (27.2%) | 3.0 ± 0.6 | 0.058 |
| No | 211 (72.8%) | 2.9 ± 0.6 |  |
| Total | 290 (100.0%) | 2.9 ± 0.6 |  |
| **Pedideal pulse** |  |  |  |
| Present | 288 (99.3%) | 2.9 ± 0.6 | 0.331 |
| Absent | 2 (0.7%) | 2.5 ± 0.8 |  |
| Total | 290 (100.0%) | 2.9 ± 0.6 |  |
| **Monofilament test** |  |  |  |
| Yes | 281 (96.9%) | 2.9 ± 0.6 | 0.878 |
| No | 9 (3.1%) | 2.9 ± 0.5 |  |
| Total | 290 (100.0%) | 2.9 ± 0.6 |  |

**Supplementary Table 2: correlation between comorbidities and PFT**

| **Obesity** | **N (%)** | **PFT (mm)** | **P value** |
| --- | --- | --- | --- |
| **Yes** | 29 (10.3%) | 3.0 ± 0.6 | 0.453 |
| **No** | 252 (89.7%) | 2.9 ± 0.6 |  |
| **Total** | 281 (100.0%) | 2.9 ± 0.6 |  |
| **Hypertension** |  |  |  |
| **Yes** | 102 (35.4%) | 3.0 ± 0.6 | **0.022** |
| **No** | 186 (64.6%) | 2.8 ± 0.6 |  |
| **Total** | 288 (100.0%) | 2.9 ± 0.6 |  |
| **Cardiovascular Disease** |  |  |  |
| **Absent** | 248 (88.3%) | 2.9 ± 0.6 | **0.018** |
| **Present** | 33 (11.7%) | 3.1 ± 0.5 |  |
| **Total** | 281 (100.0% | 2.9 ± 0.6 |  |
